# Retinal Parameters as Compared with Head Circumference, Height, Weight, and Body Mass Index in Children in Kenya and Bhutan

**DOI:** 10.4269/ajtmh.17-0943

**Published:** 2018-06-11

**Authors:** Sara J. Grundy, Lhab Tshering, Stanley W. Wanjala, Megan B. Diamond, Martin S. Audi, Sashank Prasad, Russell T. Shinohara, Debora Rogo, Dechen Wangmo, Ugyen Wangdi, Abi Aarayang, Thukten Tshering, Thomas F. Burke, Farrah J. Mateen

**Affiliations:** 1 Neurological Clinical Research Institute, Massachusetts General Hospital, Boston, Massachusetts;; 2 Department of Psychiatry, Jigme Dorji Wangchuck National Referral Hospital, Thimphu, Bhutan;; 3 Department of Social Sciences, Pwani University, Kilifi, Kenya;; 4 Sagam Community Hospital, Siaya, Kenya;; 5 Harvard Medical School, Boston, Massachusetts;; 6 Division of Neuro-Ophthalmology, Brigham and Women’s Hospital, Boston, Massachusetts;; 7 Department of Biostatistics, University of Pennsylvania, Philadelphia, Pennsylvania;; 8 Department of Ophthalmology, Jigme Dorji Wangchuck National Referral Hospital, Thimphu, Bhutan;; 9 Division of Global Health and Human Rights, Massachusetts General Hospital, Boston, Massachusetts;; 10 Harvard T.H. Chan School of Public Health, Boston, Massachusetts

## Abstract

The retina shares embryological derivation with the brain and may provide a new measurement of overall growth status, especially useful in resource-limited settings. Optical coherence tomography (OCT) provides detailed quantification of retinal structures. We enrolled community-dwelling children ages 3–11 years old in Siaya, Kenya and Thimphu, Bhutan in 2016. We measured head circumference (age < 5 years only), height, and weight, and standardized these by age and gender. Research staff performed OCT (*iScan*; Optovue, Inc., Fremont, CA), measuring the peripapillary retinal nerve fiber layer (RNFL) and macular ganglion cell complex (GCC) thicknesses. A neuro-ophthalmologist performed quality control for centration, motion artifact, and algorithm-derived quality scores. Generalized estimating equations were used to determine the relationship between anthropometric and retinal measurements. Two hundred and fifty-eight children (139 females, average age 6.4 years) successfully completed at least one retinal scan, totaling 1,048 scans. Nine hundred and twenty-two scans (88.0%) were deemed usable. Fifty-three of the 258 children (20.5%) were able to complete all six scans. Kenyan children had a thinner average GCC (*P* < 0.001) than Bhutanese children after adjustment for age and gender, but not RNFL (*P* = 0.70). In models adjusting for age, gender, and study location, none of standardized height, weight, and body mass index (BMI) were statistically significantly associated with RNFL or GCC. We determined that OCT is feasible in some children in resource-limited settings, particularly those > 4 years old, using the *iScan* device. We found no evidence for GCC or RNFL as a proxy for height-, weight-, or BMI-for-age. The variation in mean GCC thickness in Asian versus African children warrants further investigation.

## INTRODUCTION

Few growth parameters exist to measure sensory organ function in children in resource-limited settings. Height and weight are often used to monitor growth status, but these measurements offer a gross overall assessment. More precise measurements are needed for sensorineural development, particularly in settings where examining equipment may be antiquated or inaccessible. Retinal development continues until to age 4 years of life,^1^ suggesting that retinal nerve fiber layer (RNFL) measurements and other retinal parameters may be objective quantitative markers of early life growth and development.

Embryologically, the retina shares derivation with the cerebral cortex,^1^ but unlike the cortex, it can be visualized noninvasively. Optical coherence tomography (OCT) is an emerging technology that provides detailed high-resolution quantification and cross-sectional visualization of ocular structures, including the retina.^2^ Optical coherence tomography is portable, painless, noninvasive, and easy to operate. Its value in resource-limited settings has yet to be studied but its properties make it an ideal instrument for the understanding of retinal parameters in children.

Optical coherence tomography–based measurement of retinal layers has been performed in children in high-income settings and shows correlations with prematurity, low birth weight, and ethnicity.^3–7^ These findings require confirmation across populations and regions since “normative” data may be influenced by a variety of factors that have yet to be fully understood. Optical coherence tomography has also been studied in the diagnosis of specific ocular disorders of childhood, including pediatric glaucoma, retinopathy of prematurity, and shaken baby syndrome,^8–10^ yet normative data on retinal parameters in children are limited^11^ and do not exist for children in non-Western settings or in sub-Saharan Africa.

Here we assess OCT for its feasibility of use in community-dwelling children in two lower income countries. We compare OCT parameters with standard growth measurements to explore the use of this emerging technology in resource-limited settings. Through this work, we establish normative data for retinal parameters by age, gender, and location in these lower income pediatric populations as a foundation for future comparative research.

## MATERIALS AND METHODS

### Ethics approvals and protocols.

The study protocol was approved by the Research Ethics Board of the Ministry of Health of the Kingdom of Bhutan, the Maseno University Ethics Review Committee (Kenya), and the Partners Healthcare Institutional Review Board (United States). Consent was provided by a parent, or the next of kin when a parent was not available, in the spoken language (Swahili or Luo in Kenya and Dzongkha or English in Bhutan). A thumbprint was used in place of a signature when the parent or guardian was illiterate.

### Setting.

Siaya is a rural county located in the western region of Kenya, a low-income country in sub-Saharan Africa. Siaya has a population of 843,304.^12^ Recruitment took place at nine primary and secondary schools and one orphanage in Siaya between January and March 2016.

The Kingdom of Bhutan is a lower middle-income country in Asia, located between China and India. Recruitment centered at Jigme Dorji Wangchuck National Referral Hospital (JDWNRH) between August and October 2016. The Jigme Dorji Wangchuck National Referral Hospital is located in the capital city of Thimphu (population 128,342).^13^

### Participants.

Community-dwelling children up to 11 years old were eligible for study inclusion. Children were enrolled if their age was known and they could successfully complete at least one scan. Children were excluded if they were blind in one or both eyes or had a history of eye trauma.

### Enrollment.

To recruit participants in Kenya, the project manager, research assistant, and local optometrist approached the school’s headmaster and asked for permission to conduct the study in the school. An information session was then conducted in Swahili and the local language, Luo. The local population speaks both Swahili and Luo, although Swahili and English are the primary written languages.

In Bhutan, participants were recruited from the Departments of Ophthalmology and Pediatrics at JDWNRH. Participants presented for routine vision screenings, general pediatric “checkup” visits, or accompanied their parents who were receiving outpatient care elsewhere in the hospital. Pediatric participants were not seeking care for neurological or ophthalmological issues directly.

### Questionnaire.

Specific demographic and clinical variables of interest included the child’s age, gender, educational level, self-reported human immunodeficiency virus (HIV) status, known medical conditions, and perinatal history (Supplemental Appendix 1).

### Anthropometric measurements.

Each child was measured for current clothed height (cm), weight (kg), and head circumference (cm). Body mass index (BMI) was calculated for each child in the standard manner (weight [kg]/height[m]^2^). Head circumference was measured using the standard procedure of the Centers for Disease Control and Prevention.^14^ Data were collected by two research staff in the same way for every child at each location. Age and gender standardized *Z* scores were calculated for each child using the methods and growth standards of the World Health Organization.^15^ Standardized growth charts and *Z* scores were only available for children younger than 5 years old for head circumference. Z scores were dichotomized as ≥ 0 for all analyses.

### Equipment and eye scan procedures.

All OCT scans and anthropometric measurements were performed on-site with the exception of one school in Kenya that did not have electricity. In the latter situation, the OCT scans and measurements were conducted in the Optometry Office at Sagam Community Hospital.

**Figure 1.**
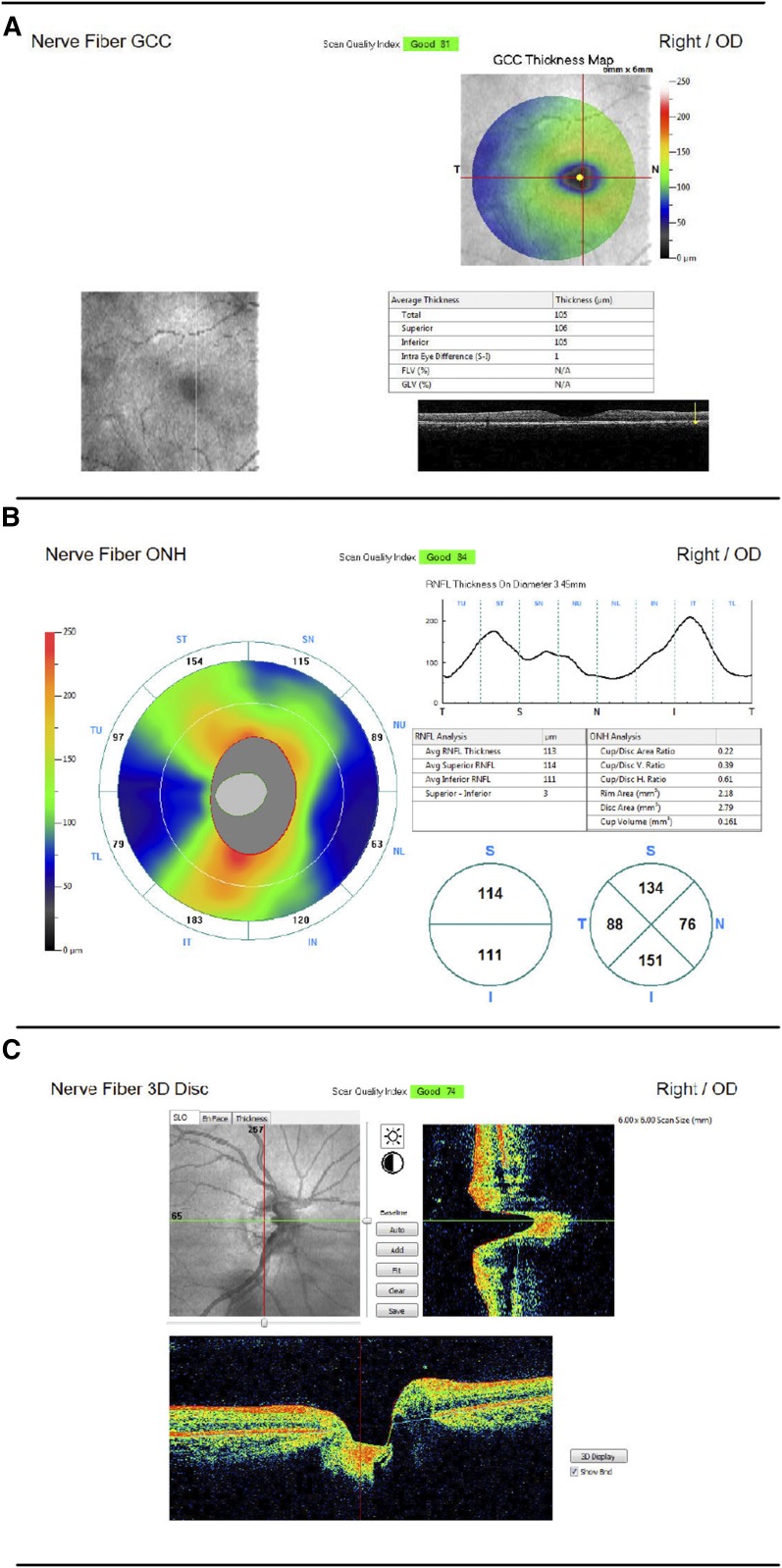
Sample scan output. (**A**) Nerve fiber ganglion cell complex (GCC), (**B**) nerve fiber optic nerve head (ONH), (**C**) nerve fiber 3D disc. RNFL = retinal nerve fiber layer. This figure appears in color at www.ajtmh.org.

Optical parameters were measured using an *iScan* OCT machine (Optovue, Inc.). All scans were conducted by a nonphysician project manager or research assistant who received OCT training at the Massachusetts Eye and Ear Infirmary or JDWNRH. Each child sat for six scans (three of each eye): the nerve fiber optic nerve head (ONH), the nerve fiber ganglion cell complex (GCC), and nerve fiber three dimensional (3D) disc scans. Data obtained from GCC scans included the average thickness of the GCC layer as well as the superior and inferior portions separately. Optic nerve head measurements included the average thickness of the RNFL layer over all quadrants of the retina and thicknesses of each quadrant individually (superior, inferior, nasal, and temporal). The 3D disc scan mapped the optic disc to measure optic disc and optic cup dimensions and ratios (Figure 1). The order of scans was the same for each participant beginning with GCC, followed by ONH, and 3D Disc. Ganglion cell complex and ONH scans took less than 1 second each and 3D disc scans required approximately 2 seconds to complete.

Retinal nerve fiber layer thickness is mapped from the optic disc margin to 4.93 mm from the disc center. Retinal nerve fiber layer thicknesses are measured at a diameter of 3.45 mm and reported at eight radial points and graphically displayed. The disc margin used as a reference is mapped using the nerve fiber 3D disc scan. For GCC scans, the thinnest portion is detected to center the scan at the fovea. The *iScan* OCT device has a resolution of 5 μm, a scan range depth of 2.3 mm, and a scan speed of 26,000 A-scans/second for the human retina.^16^

### Quality control.

All scans were reviewed for quality by a Boston-based neuro-ophthalmologist. Each scan contained a scan quality index score out of 100. Scan quality cutoffs were determined a priori. Scores below 40 for nerve fiber ONH and nerve fiber 3D disc scans and below 32 for nerve fiber GCC scans were not used in the final analysis. These values are set as the quality threshold by Optovue as the signal and clarity levels required to distinguish critical retinal layers.^16^ Scans were also qualitatively reviewed for centration, presence of floaters, movement artifacts, or other scan abnormalities.^16^ Nerve fiber 3D disc scans were reviewed for disconnected or distorted blood vessel patterns, which indicate a motion artifact.

### Statistical analysis.

Exploratory data analyses included the construction of histograms and scatterplots of RNFL thickness, GCC thickness, and anthropometric variables. In cases where more than one measurement of RNFL thickness or GCC thickness was recorded because of quality issues with initial acquisitions in a participant, only final measurements were used. Paired *t*-tests were used to confirm that no mean differences were present between the two eyes. Generalized estimating equation-based models were separately fit on RNFL thickness and GCC thickness to assess the relationship with each metric separately using an exchangeable correlation structure and adjusting for age and gender. Participants with missing measurements for particular variables were excluded only in analyses involving those variables to maximize statistical power. All statistical analyses were conducted in the *R* statistical environment (Vienna, Austria) (https://www.R-project.org/). All hypothesis testing was conducted using two-sided tests and a significance level of *P* < 0.05.

## RESULTS

### Participant demographic characteristics and health history.

Two hundred and fifty-eight community-dwelling children (average age 6.4 years, 53.9% female) were enrolled, including 130 children from Bhutan and 128 from Kenya. The demographic characteristics of the two populations were similar, including age, gender, and level of education (Table 1).

**Table 1:** Demographic characteristics

	Kenya *n* (%)	Bhutan *n* (%)	Total *n* (%)
No. of participants	128 (49.6)	130 (50.4)	258
Age, years ± SD	6.3 ± 1.4	6.5 ± 1.5	6.4 ± 1.5
No. female	69 (53.9)	70 (53.8)	139 (53.9)
Years in school			
None	2 (1.7)	26 (20.8)	28 (10.9)
≤ 6 months	8 (6.9)	5 (4.0)	13 (5.0)
7–12 months	1 (0.9)	13 (10.4)	14 (5.4)
12–24 months	10 (8.6)	27 (21.6)	37 (14.3)
> 24 months	95 (81.9)	54 (43.2)	149 (57.8)
Medical complications			
Pregnancy complications	12 (13.5)	7 (5.6)	19 (7.4)
Pregnancy hospitalizations	6 (6.7)	13 (10.4)	19 (7.4)
Child medical problems	43 (48.3)	14 (11.4)	46 (17.8)
HIV			
Infected	7 (7.9)	1 (0.9)	8 (3.1)
Uninfected	60 (67.4)	112 (99.1)	172 (66.7)
Unknown	22 (24.7)	0 (0.0)	22 (8.5)

SD = standard deviation.

Perinatal and pregnancy complications of the mother were reported more frequently for Kenyan children than Bhutanese children (13.5% versus 5.6%) as were medical problems among the children (48.3% versus 11.4%). A wide range of current and previous pediatric medical problems were reported including sickle cell anemia, asthma, and nephrolithiasis as well as history of prior pneumonia, malaria, and meningitis (Supplemental Appendix 2). HIV was self-reported in seven (7.9%) children in Kenya and one child in Bhutan (0.9%).

### Anthropometric measurements.

Anthropometric measurements including height, weight, BMI, and head circumference were available for 123 (96%) Kenyan children and 101 (78%) Bhutanese children. Data were missing for five children in Kenya. In Bhutan, the first 29 enrolled children did not undergo measurements because of logistical issues only.

Results for height, weight, and BMI were skewed for both locations, more so for the children in Kenya. In Bhutan, 64 (63.4%) children had *Z* scores below 0 for BMI-for-age, 56 (55.4%) for height-for-age, and 67 (65.0%) for weight-for-age. In Kenya, 104 (84.6%) children had *Z* scores below 0 for BMI-for-age, 88 (71.5%) for height-for-age, and 87 (70.7%) for weight-for-age. Results for head circumference-for-age were normally distributed for children in both locations with 10 (45.5%) children with *Z* scores below 0 in Kenya and 11 (50.0%) children in Bhutan (Table 2).

**Table 2:** Standardized growth parameters by study location

	Kenya	Bhutan	Total
Height-for-age			
< −3	9 (7.3)	–	9 (4.0)
> −3 and < −2	9 (7.3)	5 (5.0)	14 (6.3)
> −2 and < −1	28 (22.8)	15 (14.9)	43 (19.2)
> −1 and < 0	42 (34.1)	36 (35.6)	78 (34.8)
> 0 and < 1	27 (22.0)	33 (32.7)	60 (26.8)
> 1 and < 2	5 (4.1)	10 (9.9)	16 (6.7)
> 2 and < 3	2 (1.6)	2 (2.0)	4 (1.8)
> 3	1 (0.8)	–	1 (0.4)
Weight-for-age			
< −3	3 (2.4)	1 (1.0)	4 (1.8)
> −3 and < −2	6 (4.9)	4 (3.9)	10 (4.4)
> −2 and < −1	30 (24.4)	21 (20.4)	51 (22.6)
> −1 and < 0	48 (39.0)	41 (39.8)	89 (39.4)
> 0 and < 1	26 (21.1)	28 (27.2)	54 (23.9)
> 1 and < 2	9 (7.3)	5 (48.5)	14 (6.2)
> 2 and < 3	1 (0.8)	3 (2.9)	4 (1.8)
> 3	–	–	–
BMI-for-age			
< −3	2 (1.6)	2 (2.0)	4 (1.8)
> −3 and < −2	7 (5.7)	5 (5.0)	12 (5.4)
> −2 and < −1	71 (5.8)	22 (21.8)	93 (41.5)
> −1 and < 0	24 (19.5)	35 (34.7)	59 (26.3)
> 0 and < 1	15 (12.2)	27 (26.7)	42 (18.8)
> 1 and < 2	4 (3.3)	7 (6.9)	11 (4.9)
> 2 and < 3	–	3 (3.0)	3 (1.3)
> 3	–	–	–
Head circumference-for-age			
< −3	–	–	–
> −3 and < −2	–	–	–
> −2 and < −1	3 (13.0)	4 (18.2)	7 (15.6)
> −1 and < 0	7 (30.4)	7 (31.8)	14 (31.1)
> 0 and < 1	8 (34.8)	6 (27.3)	14 (31.1)
> 1 and < 2	4 (17.4)	3 (13.6)	7 (15.6)
> 2 and < 3	1 (4.3)	2 (9.1)	3 (6.7)
> 3	–	–	–

BMI = body mass index.

Children who are at least two standard deviations below the mean for a height or weight measurement are considered stunted or pathologically underweight, respectively. In Bhutan, seven (7.0%) children had *Z* scores below −2 for BMI-for-age, five (5.0%) for height-for-age, and five (4.9%) for weight-for-age. In Kenya, nine (7.3%) children had *Z* scores below −2 for BMI-for-age, 18 (14.6%) for height-for-age, and nine (7.3%) for weight-for-age. There were no children in either location with a head circumference *Z* score below −2.

### Feasibility.

There were 1,048 completed scans from 258 children. Following quality control, 922 (88.0%) scans were usable. This resulted in a final scan success rate of 59.6% of the initial target goal of 1,548 scans, i.e., six scans from each participant. Only 53 (20.5%) of children were able to successfully complete all six scans. Many of the children, particularly those at younger ages had difficulty completing the scans, which led to a large degree of missing data. The degree of missingness was strongly associated with age, with younger aged children most likely to have missing data (*P* < 0.001). This was the case for five of six scan types, excluding GCC in the right eye, which was the first scan chronologically. The proportion of completed scans varied by scan type as well. Across all ages, GCC scans had the highest completion proportion of 84.1%. The completion percentages for nerve fiber ONH and nerve fiber 3D disc scans were lower at 57.2% and 37.4%, respectively (Table 3).

**Table 3:** Feasibility by age, years

	3	4	5	6	7	8	9	10	Total
Total number of eyes	16	64	90	154	112	56	20	4	516
Nerve fiber ONH	1 (6.3)	17 (26.6)	45 (50.0)	92 (59.7)	74 (66.1)	43 (76.8)	19 (95.0)	4 (100.0)	295 (57.2)
Nerve fiber GCC	9 (56.3)	49 (76.6)	73 (81.1)	137 (89.0)	95 (84.8)	49 (87.5)	18 (90.0)	4 (100.0)	434 (84.1)
Nerve fiber 3D disc	0 (0.0)	13 (20.3)	28 (31.1)	60 (39.0)	51 (45.5)	27 (48.2)	11 (55.0)	3 (75.0)	193 (37.4)

GCC = ganglion cell complex; ONH = optic nerve head.

### Retinal parameters.

A total of 295 ONH scans and 464 GCC scans were obtained from 258 children. The mean RNFL and GCC thicknesses and standard deviations were calculated for all 258 children and also stratified by age, gender, and location to produce a normative dataset for these retinal parameters in children (Supplemental Table 1).

The mean RNFL thickness for all participants was 108.3 ± 10.0 μm in the right eye and 108.6 ± 11.5 μm in the left eye. The mean GCC thickness was 98.0 ± 6.4 μm in the right eye and 98.4 ± 6.0 μm in the left eye. When testing for a difference in measurements between right and left eyes, the *P* value was 0.09. There were no statistical differences by age or gender for both RNFL and GCC measurements. Kenyan children had significantly thinner GCC measurements than Bhutanese children (*P* < 0.001), but there was no significant difference in RNFL between locations (*P* = 0.70). In a model, after adjustment for age, gender, and location, there was no observed statistically significant relationship between head circumference, BMI, height, or weight, with either RNFL or GCC. No evidence was found that OCT could act as a surrogate measure for growth status, low weight-for-age, or stunting. Therefore, the null hypothesis was accepted.

## DISCUSSION

Our data contribute to the scarcity of eye data in children in resource-limited settings by providing detailed retinal measurements in a high number of community-dwelling children, including average RNFL and GCC thicknesses categorized by age, gender, and location. This work, in spite of logistical challenges in completion of OCT scans, is the first to our knowledge to provide normative data for children in a sub-Saharan African setting in particular. There are currently no established normative OCT databases for children in any setting; nor are there considerations of differences by ethnicity in normative databases for adults. Our data provide a starting point for future work comparing retinal parameters in children in resource-limited settings.

Mitosis in the retina primarily occurs during gestation, yet cell differentiation and other developmental processes continue through the first 4 years of life.^1^ Previous studies have shown premature birth and low birth weight to have lasting effects on retinal development that are detectable both at birth and later in life.^5,17,18^ One study of 50 term infants and 57 preterm infants born before 30 weeks found that average RNFL thickness was decreased in preterm infants when measured at 36 weeks compared with term births.^17^ In a separate study of 25 preterm children and 54 full-term controls, RNFL was measured at the average age of 10 years old and found to be 9–13% thinner in those children born preterm.^18^ These differences were also found in a study of 12 adults, where abnormal foveal structure was detected using OCT at an average age of 39 years.^4^ We hypothesized that a positively associated relationship would exist between retinal parameters and current growth metrics, because research has shown reduced RNFL thickness resulting from preterm birth and low birth weight with the impact detectable into adulthood^3–5,17,19,20^ However, despite this putative impact, we found that current head circumference, height, weight, and BMI were not associated with a difference in RNFL or GCC thickness.

It is possible that our measurements occurred too late in life for important differences to be observed in the retina or that there is truly no impact on the retina in children with delayed overall growth. Because retinal integrity is extremely important for vision, the retinal structures may have preferential use of the body’s nutrients when nutrition is limited. This remains hypothetical because our data were not longitudinally collected, and could not start at the time of birth because of technical limitations of the OCT device. Retinal development is believed to cease by an age of 4 years, and the average age of children in this study was 6.4 years old. This places most participants past the timeframe when many visual developmental processes are thought to occur.^1^

In addition, our results showed that the Kenyan children had statistically significant thinner GCC (*P* < 0.001) than the Bhutanese children. Although we cannot confirm that these two populations differ in GCC thickness because of race, our findings are consistent with one study that found African Americans had thinner fovea as compared with Caucasians,^21^ although this study was conducted primarily in adults. Limited research has been carried out to determine differences in retinal parameters by race in children. One study on 2,367 12-year-olds in Australia found that South Asian children had thinner inner, central, and outer macula compared with Caucasian, East Asian, and Middle Eastern children.^22^ A separate study of 4,118 6–12-year-olds found East Asian children to have thicker RNFL measurements than Caucasian children.^23^ Neither of these studies included children of African descent, providing no direct comparison between African and Asian children. One study of 146 healthy African-American children 6–17 years old measured ONH parameters but did not include other racial or ethnic groups for comparison.^24^ Retinal nerve fiber layer has been reported to be thicker in adults of African descent compared with Caucasians^25^ although this was not reflected in our data on children.

Another potential cause of this difference may be the prevalence of infectious disease retinopathies such as in cerebral malaria^26–28^ and HIV,^29,30^ both of which are more common in Kenya than Bhutan.^31,32^ One study conducted in India found 48% of children with cerebral malaria had retinopathy at the time of diagnosis.^33^ Given the prevalence of pathogens that may infect children during development in sub-Saharan Africa and the location of previous research on retinal parameters in children in high-income countries, further investigation into geographical differences is needed to appreciate these possible influences on retinal parameters beyond physical growth.

Participants < 5 years old had difficulty completing the stationary OCT, and children < 3 years old were unable to participate entirely. The *iScan* OCT machine used in this study is designed for adult use, and therefore, the position of the pupil target and headrest led to difficulty in scan acquisition and some reduction of scan quality. In addition, participants were required to sit still and keep their eyes fixated on a green light, which was a difficult task for younger children. Because of these feasibility issues, we were able to collect only limited data for children in the youngest age groups. Handheld OCT devices may be better suited to collect data in these youngest age groups.^34^

Our study had several notable limitations. Given the setting and circumstances, we were unable to record an eye examination in the participants. Participants’ medical histories were provided by the parents in a self-report fashion and were at times inaccurate or incomplete; health awareness is generally low, for example, for a history of malaria, and a formal medical records system did not exist. Future research on retinal parameters in children in resource-limited settings would benefit from a more detailed tracking of prematurity and other birth complications, as this is the most common use of the technology in higher income settings in children. In addition, we did not confirm the diagnosis of HIV, and our sample size of HIV-infected children was too low to measure the impact of HIV infection on RNFL and GCC measurements. Finally, although we assume that there are important differences in GCC between the two populations, we are unable to explain whether these differences are because of operational issues, macronutrient or micronutrient deficiencies, infectious disease related retinopathies, genetic causes, general growth limitations, within normal variation, or a spurious finding.

Our study also had several strengths. The *iScan* by Optovue represents the most recent commercially available technology for retinal imaging. These data derive from logistically challenging environments in Africa and Asia: locations with inconsistent electricity, school attendance, and health awareness. It is precisely in these populations where objective screening measurements could have the most value for young populations. We also include a community-dwelling sample. Although it is not an enumerated, population-based sample, it reflects a typical group of children in the targeted populations. We also used quality control metrics, both designated by the company in its automated machine algorithms and by a review by a neuro-ophthalmologist who examined other aspects of scan quality that may be missed by automation.

Despite the lack of a relationship between OCT-based measures of retinal thickness and anthropometric measurements, OCT remains valuable for screening and diagnosing ocular disorders including glaucoma, which impact children and adults.^8^ Future directions for the use of OCT in sub-Saharan Africa and Central Asia include the measurement of retinal parameters in younger children, including from the neonatal period to primary school age. To do this, hardware improvements to the OCT machine specifically for use in children would be necessary. Correlation with health status in other ways, such as serum testing for vitamin and micronutrient levels, as well as common infectious diseases, would also aid exploration of the potential etiologies of variations in retinal parameters.

## Supplementary Material

Supplemental appendices
