# Supplementary material for: Retinal Parameters as Compared with Head Circumference, Height, Weight, and Body Mass Index in Children in Kenya and Bhutan
Source: Am J Trop Med Hyg. 2018 Jun 11;99(2):482–8. doi: 10.4269/ajtmh.17-0943 (PMC6090321; doi:10.4269/ajtmh.17-0943)
Supplement: Supplementary file 1 [file tpmd170943.SD1.pdf]

SUPPLEMENTAL APPENDIX 1

OPTICAL PARAMETERS FOR BRAIN DEVELOPMENT PARTICIPANT SURVEY

Participant code: ___________________________________
Date: ____
Guardian (parent, next of kin etc)
Name: ____
Relationship to child: Parent/guardian specify: __________
Languages: ____
Phone number: ____
Address: ____

____

Child
Name: ____
Age (years, months): _______________ Date of birth: ____________________
Gender: Male Female.
Languages: ____
Participant code: ___________________________________

Characteristics
Years of school completed: No school 0–6 months 6–12 months 1–2 years 2 years+
HIV status: Known positive Nonreactive Unknown.
Did the participant's mother have any medical problems during pregnancy (preeclampsia, gestational diabetes, bleeding, and early contractions): Yes No Unknown
If yes, describe:
Was the participant's mother hospitalized before or after the pregnancy?
Yes No Unknown.
If yes, what was she hospitalized for:
Does the child have any other medical conditions (*If none, write none. If unknown, write UNK***):?**

Anthropometric measurements
Height (cm): Weight (kg): Head circumference (cm):
Comments:

SUPPLEMENTAL APPENDIX 2

Kenya
Current or previous malaria n = 12
Itchy/watery/red eyes n = 7
Persistent cough n = 4

Three or fewer children reported the following conditions:
Ear infection/pain
Stomach pains
Asthma
Jigger (*Tunga penetrans*) infection
Sores
Sickle cell anemia
Previous sepsis
Headaches
Diarrhea
Scabies
Renal incontinence
Rash
Anal warts
Vocal cord issue
Chest pain

Nose bleeds
Opportunistic infections

Bhutan

Three or fewer children reported the following conditions:
Headaches/migraine
Previous pneumonia
Previous meningitis
Kidney stones
Ear pain
Leg pain
Dental caries

Supplemental Table 1

Retinal parameters

		Age, years	3	4	5	6	7	8	9	10	11	Total
All												
	OD	Average RNFL, μm	NA	109.7 ± 11.9	108.3 ± 13.0	107.1 ± 8.3	108.5 ± 10.7	109.2 ± 10.9	107.1 ± 7.8	111.6 ± 7.3	110 (*N* = 1)	108.3 ± 10.0
		Superior	NA	113.4 ± 14.5	109.6 ± 15.5	108.2 ± 10.4	110.1 ± 12.8	110.0 ± 12.7	111.1 ± 6.5	114.6 ± 6.5	111 (*N* = 1)	109.8 ± 11.9
		Inferior	NA	106.6 ± 11.2	107.2 ± 12.7	106.2 ± 8.5	107.3 ± 10.3	108.2 ± 11.1	103.1 ± 10.9	109.2 ± 8.8	109 (*N* = 1)	106.9 ± 10.1
		Nasal	NA	89.3 ± 13.9	86.6 ± 14.3	84.3 ± 13.8	87.3 ± 17.7	85.3 ± 20.3	80.5 ± 9.7	96.4 ± 17.4	86 (*N* = 1)	85.9 ± 16.1
		Temporal	NA	71.3 ± 8.2	79.6 ± 9.1	80.1 ± 11.0	77.7 ± 13.0	84.1 ± 18.3	85.2 ± 10.5	74.0 ± 7.8	84 (*N* = 1)	79.8 ± 13.0
		Average GCC, μm	92.0 ± 6.6	98.3 ± 6.4	95.8 ± 8.4	98.3 ± 5.8	98.7 ± 5.8	98.8 ± 6.3	97.2 ± 5.7	100 ± 3.9	97 (*N* = 1)	98.0 ± 6.4
		Superior	94.0 ± 6.2	97.8 ± 6.6	95.1 ± 8.6	97.4 ± 6.0	97.7 ± 5.9	98.6 ± 6.8	96.8 ± 5.5	99.3 ± 3.6	96 (*N* = 1)	97.3 ± 6.6
		Inferior	89.3 ± 6.5	99.0 ± 6.7	96.0 ± 8.6	98.5 ± 6.8	99.7 ± 5.9	99.4 ± 6.0	98.9 ± 6.1	100.8 ± 5.1	99 (*N* = 1)	98.5 ± 6.9
	OS	Average RNFL, μm	114 (*N* = 1)	104.3 ± 9.3	109.2 ± 9.7	107.3 ± 10.9	110.2 ± 10.5	108.7 ± 9.6	106.3 ± 8.4	110.8 ± 5.0	112 (*N* = 1)	108.6 ± 10.0
		Superior	114 (*N* = 1)	106.5 ± 9.7	112.8 ± 10.8	110.1 ± 10.6	113.1 ± 11.4	111.2 ± 8.3	108.6 ± 10.6	115.8 ± 7.0	119 (*N* = 1)	111.5 ± 11.6
		Inferior	115 (*N* = 1)	101.8 ± 9.7	106.2 ± 10.8	104.7 ± 10.6	107.0 ± 11.4	106.2 ± 8.3	103.9 ± 10.6	106.4 ± 7.0	106 (*N* = 1)	105.7 ± 10.2
		Nasal	70 (*N* = 1)	77.5 ± 9.8	86.3 ± 19.8	83.5 ± 11.5	87.4 ± 14.5	84.1 ± 15.0	80.6 ± 9.2	84.4 ± 12.7	82 (*N* = 1)	84.5 ± 14.0
		Temporal	79 (*N* = 1)	74.3 ± 9.3	74.9 ± 15.7	78.2 ± 9.7	75.2 ± 8.0	76.6 ± 11.2	77.1 ± 9.5	79.6 ± 2.8	84 (*N* = 1)	76.6 ± 10.2
		Average GCC, μm	90.0 ± 4.2	97.5 ± 5.1	97.7 ± 5.9	98.6 ± 6.0	99.3 ± 5.9	98.5 ± 6.1	97.4 ± 7.4	101.0 ± 4.2	96 (*N* = 1)	98.4 ± 6.0
		Superior	89.0 ± 6.4	97.1 ± 5.4	98.1 ± 6.2	98.9 ± 5.9	99.0 ± 5.4	98.8 ± 6.1	97.1 ± 8.0	101.3 ± 4.2	99 (*N* = 1)	98.5 ± 6.3
		Inferior	90.5 ± 6.4	97.5 ± 5.4	97.5 ± 6.2	98.4 ± 5.9	99.2 ± 5.4	98.1 ± 6.1	97.4 ± 8.0	101.0 ± 4.2	93 (*N* = 1)	98.2 ± 6.0
Bhutan												
	OD	Average RNFL, μm	NA	115.0 ± 9.9	107.6 ± 18.9	108.1 ± 8.9	110.1 ± 13.0	107.5 ± 12.2	106.0 ± 6.4	111.0 ± 9.2	NA	108.6 ± 11.5
		Superior	NA	124.0 ± 12.7	102.4 ± 22.5	109.6 ± 12.4	113.6 ± 16.4	108.8 ± 14.4	111.0 ± 6.7	115.7 ± 7.5	NA	110.6 ± 14.5
		Inferior	NA	106.0 ± 7.1	112.8 ± 16.9	106.9 ± 9.8	108.0 ± 11.9	106.1 ± 11.8	101.0 ± 7.6	107.3 ± 11.5	NA	106.9 ± 11.2
		Nasal	NA	102.5 ± 16.3	83.0 ± 8.2	78.8 ± 10.8	88.7 ± 20.4	80.5 ± 12.9	76.8 ± 7.9	98.7 ± 19.8	NA	83.8 ± 15.2
		Temporal	NA	65.5 ± 12.0	81.6 ± 9.5	82.8 ± 13.7	75.5 ± 14.3	83.2 ± 12.9	85.3 ± 9.6	73.0 ± 8.7	NA	79.8 ± 12.9
		Average GCC, μm	92.0 ± 6.6	96.4 ± 6.0	95.0 ± 9.6	97.5 ± 4.5	96.8 ± 5.3	98.4 ± 5.8	96.3 ± 5.7	98.7 ± 3.5	NA	96.7 ± 6.5
		Superior	94.0 ± 6.2	95.9 ± 6.5	94.5 ± 9.4	97.4 ± 5.6	96.4 ± 5.3	98.5 ± 6.4	96.1 ± 5.9	98.3 ± 3.8	NA	96.5 ± 6.7
		Inferior	89.3 ± 6.5	95.8 ± 6.4	94.9 ± 9.5	96.3 ± 4.9	97.5 ± 5.4	98.3 ± 5.5	98.2 ± 5.4	99.0 ± 4.6	NA	96.5 ± 6.6
	OS	Average RNFL, μm	114 (*N* = 1)	NA	113.3 ± 8.8	110.4 ± 10.7	110.5 ± 9.7	105.9 ± 9.3	104.0 ± 7.6	108.7 ± 5.7	NA	108.8 ± 9.2
		Superior	114 (*N* = 1)	NA	116.6 ± 6.7	113.5 ± 7.0	115.4 ± 12.4	107.3 ± 8.4	105.4 ± 8.8	114.0 ± 8.3	NA	111.7 ± 12.1
		Inferior	115 (*N* = 1)	NA	111.4 ± 6.7	107.5 ± 7.0	105.2 ± 12.4	104.5 ± 8.4	102.6 ± 8.8	103.7 ± 8.3	NA	106.1 ± 9.0
		Nasal	70 (*N* = 1)	NA	95.7 ± 22.6	82.4 ± 12.9	81.4 ± 10.7	82.3 ± 18.0	76.8 ± 5.0	82.7 ± 9.3	NA	83.0 ± 15.3
		Temporal	79 (*N* = 1)	NA	73.0 15.7	75.6 ± 14.5	74.4 ± 9.1	75.7 ± 9.7	77.6 ± 10.5	79.7 ± 3.1	NA	75.6 ± 10.9
		Average GCC, μm	90.0 ± 4.2	96.8 ± 4.4	96.8 ± 5.6	96.8 ± 5.7	97.7 ± 4.5	98.3 ± 0.3	94.7 ± 5.2	100.3 ± 4.9	NA	97.1 ± 5.3
		Superior	89.0 ± 6.4	96.5 ± 4.7	97.0 ± 6.1	96.9 ± 5.2	97.8 ± 4.6	98.7 ± 5.4	94.6 ± 5.9	100.7 ± 4.9	NA	97.2 ± 5.5
		Inferior	90.5 ± 6.4	97.2 ± 4.6	96.7 ± 6.1	96.7 ± 5.2	97.6 ± 4.6	97.8 ± 5.4	94.4 ± 5.9	100.3 ± 4.9	NA	96.9 ± 5.3
Kenya												
	OD	Average RNFL, μm	NA	107.6 ± 13.0	108.6 ± 11.0	106.8 ± 8.3	107.6 ± 9.6	111.0 ± 9.4	109.0 ± 11.1	112.5 ± 6.4	110 (*N* = 1)	108.1 ± 9.2
		Superior	NA	109.2 ± 14.0	112.3 ± 12.0	107.8 ± 10.0	108.3 ± 10.4	111.2 ± 11.0	111.3 ± 7.5	113 ± 7.1	111 (*N* = 1)	109.3 ± 10.4
		Inferior	NA	106.8 ± 13.2	105.1 ± 10.7	106.0 ± 8.3	106.9 ± 9.6	110.5 ± 10.2	106.7 ± 16.5	112 ± 4.2	109 (*N* = 1)	106.9 ± 9.5
		Nasal	NA	84.0 ± 10.2	88.2 ± 16.3	85.9 ± 14.3	86.5 ± 16.7	91.3 ± 26.4	86.7 ± 10.7	93.0 ± 19.8	86 (*N* = 1)	87.2 ± 16.6
		Temporal	NA	73.6 ± 6.3	78.6 ± 9.2	79.4 ± 10.2	78.9 ± 12.4	85.1 ± 23.7	85.0 ± 13.5	75.5 ± 9.2	84 (*N* = 1)	79.8 ± 13.0
		Average GCC, μm	NA	100.1 ± 6.4	97.2 ± 5.9	98.8 ± 6.5	100.3 ± 5.8	99.4 ± 7.1	99.3 ± 6.4	104 (*N* = 1)	97 (*N* = 1)	99.3 ± 6.2
		Superior	NA	99.3 ± 6.6	96.3 ± 6.9	97.4 ± 6.3	98.9 ± 6.3	98.8 ± 7.6	98.3 ± 5.0	102 (*N* = 1)	96 (*N* = 1)	98.1 ± 6.4
		Inferior	NA	102.0 ± 5.8	98.1 ± 6.1	100.0 ± 7.5	101.6 ± 5.7	101.1 ± 6.6	100.3 ± 8.3	106 (*N* = 1)	99 (*N* = 1)	100.6 ± 6.5
	OS	Average RNFL, μm	NA	104.3 ± 9.3	106.8 ± 9.7	106.7 ± 11.0	110.1 ± 10.9	112.5 ± 9.1	110.0 ± 9.8	114 ± 0	112 (*N* = 1)	108.5 ± 10.4
		Superior	NA	106.5 ± 9.7	110.6 ± 11.7	109.4 ± 11.2	112.3 ± 11.2	116.5 ± 8.0	114.0 ± 15.1	118.5 ± 0.7	119 (*N* = 1)	111.4 ± 11.4
		Inferior	NA	101.8 ± 9.7	103.1 ± 11.7	104.1 ± 11.2	107.7 ± 11.2	108.5 ± 8.0	106.0 ± 15.1	110.5 ± 0.7	106 (*N* = 1)	105.6 ± 10.8
		Nasal	NA	77.5 ± 9.8	80.6 ± 16.6	83.7 ± 11.3	89.9 ± 15.3	86.9 ± 9.0	87.0 ± 12.3	87.0 ± 21.2	82 (*N* = 1)	85.4 ± 13.2
		Temporal	NA	74.3 ± 9.3	76.3 ± 16.3	78.8 ± 8.2	12.4 ± 7.8	77.9 ± 13.5	76.0 ± 9.9	79.5 ± 3.5	84 (*N* = 1)	77.2 ± 9.9
		Average GCC, μm	NA	98.5 ± 6.7	99.4 ± 6.1	99.6 ± 6.0	100.3 ± 6.5	98.6 ± 7.3	103.7 ± 9.0	103 (*N* = 1)	96 (*N* = 1)	99.7 ± 6.3
		Superior	NA	98.0 ± 7.0	100.1 ± 6.4	99.9 ± 6.2	99.8 ± 5.8	98.8 ± 7.3	103.0 ± 9.1	103 (*N* = 1)	99 (*N* = 1)	99.7 ± 6.7

(continued)

Supplemental Table 1
Continued

	Age, years	3	4	5	6	7	8	9	10	11	Total
	Inferior	NA	98.0 ± 7.0	99.0 ± 6.4	99.3 ± 6.2	100.3 ± 5.8	98.4 ± 7.3	104.3 ± 9.1	103 (*N* = 1)	93 (*N* = 1)	99.5 ± 6.3
Male											
OD	Average RNFL, μm	NA	112.8 ± 10.8	106.1 ± 13.4	107.0 ± 8.2	107.4 ± 12.6	107.6 ± 10.4	102.3 ± 7.6	107.0 ± 8.5	110 (*N* = 1)	107.2 ± 10.3
	Superior	NA	115.8 ± 10.7	108.9 ± 18.3	107.5 ± 10.0	108.1 ± 14.3	106.8 ± 11.6	110.0 ± 7.9	113.5 ± 9.2	111 (*N* = 1)	108.6 ± 12.3
	Inferior	NA	110.3 ± 12.7	103.7 ± 9.9	106.4 ± 8.9	107.9 ± 12.4	108.1 ± 11.1	94.7 ± 8.1	101.5 ± 7.8	109 (*N* = 1)	106.2 ± 10.4
	Nasal	NA	90.5 ± 5.4	88.5 ± 18.2	86.0 ± 11.5	88.4 ± 20.0	86.5 ± 14.3	74.7 ± 7.5	100.5 ± 27.6	86 (*N* = 1)	87.3 ± 15.3
	Temporal	NA	70.8 ± 11.3	77.1 ± 6.6	78.2 ± 7.4	75.5 ± 14.6	78.1 ± 15.0	78.5 ± 12.0	71.0 ± 11.3	84 (*N* = 1)	76.6 ± 11.1
	Average GCC, μm	99 (*N* = 1)	99.9 ± 6.4	94.3 ± 9.3	99.7 ± 5.9	97.9 ± 6.0	98.2 ± 6.0	92.3 ± 1.5	97.0 ± 2.8	97 (*N* = 1)	98.1 ± 6.6
	Superior	101 (*N* = 1)	99.0 ± 6.9	93.9 ± 9.3	99.2 ± 6.2	97.1 ± 6.3	97.9 ± 6.0	93.0 ± 1.0	97.0 ± 4.2	96 (*N* = 1)	97.5 ± 6.8
	Inferior	96 (*N* = 1)	101.7 ± 6.5	94.6 ± 9.9	100.3 ± 7.1	98.4 ± 6.0	98.5 ± 6.2	93.0 ± 2.8	96.5 ± 2.1	99 (*N* = 1)	98.7 ± 7.2
OS	Average RNFL, μm	NA	109.0 ± 12.7	107.7 ± 11.1	105.7 ± 13.4	110.3 ± 11.4	107.4 ± 16.7	104.0 ± 7.2	109.5 ± 7.8	112 (*N* = 1)	107.5 ± 11.5
	Superior	NA	110.5 ± 12.0	111.4 ± 9.9	108.5 ± 13.2	113.2 ± 12.5	108.3 ± 9.4	111.0 ± 12.4	112.0 ± 8.5	119 (*N* = 1)	110.4 ± 12.9
	Inferior	NA	107.5 ± 12.0	104.0 ± 9.9	103.2 ± 13.2	106.9 ± 12.5	106.6 ± 9.4	96.7 ± 12.4	107.0 ± 8.5	106 (*N* = 1)	104.7 ± 11.6
	Nasal	NA	83.5 ± 9.2	79.2 ± 18.8	84.1 ± 11.8	87.6 ± 16.7	88.8 ± 18.4	78.3 ± 5.5	86.5 ± 9.2	82 (*N* = 1)	84.9 ± 14.4
	Temporal	NA	78.5 ± 4.9	82.5 ± 15.4	79.6 ± 11.7	76.1 ± 10.4	72.2 ± 13.8	79.0 ± 14.0	80.0 ± 4.2	84 (*N* = 1)	77.8 ± 11.9
	Average GCC, μm	NA	96.5 ± 7.7	96.8 ± 4.8	99.5 ± 6.1	98.0 ± 6.1	97.6 ± 5.6	92.3 ± 2.3	97.5 ± 0.7	96 (*N* = 1)	98.0 ± 5.8
	Superior	NA	95.5 ± 7.5	97.1 ± 4.8	99.6 ± 6.2	97.4 ± 5.5	97.8 ± 5.6	92.7 ± 4.5	97.5 ± 0.7	99 (*N* = 1)	98.0 ± 6.4
	Inferior	NA	96.8 ± 7.5	96.7 ± 4.8	99.3 ± 6.2	98.2 ± 5.5	97.4 ± 5.6	91.7 ± 4.5	97.5 ± 0.7	93 (*N* = 1)	97.9 ± 5.7
Female											
OD	Average RNFL, μm	NA	105.7 ± 14.4	110.6 ± 13.1	107.0 ± 8.8	109.4 ± 8.9	109.8 ± 11.3	110.0 ± 7.1	114.7 ± 5.9	NA	109.0 ± 9.9
	Superior	NA	110.3 ± 20.8	110.2 ± 13.3	109.1 ± 11.0	112.1 ± 11.3	111.2 ± 13.1	111.8 ± 6.3	115.3 ± 6.4	NA	110.8 ± 11.6
	Inferior	NA	101.7 ± 8.3	110.8 ± 14.7	105.4 ± 8.0	106.7 ± 8.2	108.3 ± 11.4	108.2 ± 9.5	114.3 ± 5.0	NA	107.3 ± 9.8
	Nasal	NA	87.7 ± 23.1	85.0 ± 10.8	82.7 ± 15.8	86.1 ± 15.6	84.7 ± 22.7	84.0 ± 9.8	93.7 ± 14.0	NA	84.9 ± 16.8
	Temporal	NA	72.0 ± 2.6	81.4 ± 10.7	81.7 ± 13.2	79.9 ± 11.2	86.7 ± 19.4	88.5 ± 9.5	76.0 ± 6.6	NA	82.2 ± 13.8
	Average GCC, μm	88.5 ± 3.5	95.8 ± 6.1	96.8 ± 8.1	97.0 ± 5.5	99.3 ± 5.6	99.1 ± 6.5	99.3 ± 5.6	103.0 ± 1.4	NA	97.9 ± 6.4
	Superior	90.5 ± 2.1	95.7 ± 6.3	95.9 ± 8.4	95.6 ± 5.5	98.2 ± 5.7	98.9 ± 7.2	98.4 ± 5.8	101.5 ± 0.7	NA	97.1 ± 6.6
	Inferior	86.0 ± 4.2	95.8 ± 6.2	96.9 ± 7.9	96.9 ± 6.4	100.9 ± 5.7	99.8 ± 6.0	100.6 ± 5.7	105.0 ± 1.4	NA	98.5 ± 6.7
OS	Average RNFL, μm	114 (*N* = 1)	99.5 ± 2.1	110.1 ± 9.1	108.5 ± 7.5	110.1 ± 10.1	109.4 ± 9.3	107.6 ± 9.6	111.7 ± 4.0	NA	109.2 ± 8.7
	Superior	114 (*N* = 1)	102.5 ± 2.8	113.6 ± 11.5	111.0 ± 7.3	113.1 ± 10.9	112.7 ± 8.0	107.2 ± 7.6	118.3 ± 7.8	NA	112.1 ± 10.5
	Inferior	115 (*N* = 1)	96.0 ± 2.8	107.4 ± 11.5	106.0 ± 7.3	107.0 ± 10.9	106.0 ± 8.0	108.2 ± 7.6	106.0 ± 7.8	NA	106 ± 9.0
	Nasal	70 (*N* = 1)	71.5 ± 7.8	90.5 ± 20.1	82.4 ± 11.5	87.2 ± 13.4	81.5 ± 12.6	82.0 ± 11.3	83.0 ± 16.5	NA	84.1 ± 13.8
	Temporal	79 (*N* = 1)	70.0 ± 12.7	70.8 ± 14.9	77.2 ± 7.4	74.7 ± 6.5	79.3 ± 8.9	75.8 ± 6.6	79.3 ± 2.5	NA	75.8 ± 9.0
	Average GCC, μm	90.0 ± 4.2	98.0 ± 3.7	98.4 ± 6.6	97.3 ± 5.8	100.2 ± 5.7	99.0 ± 6.5	99.6 ± 7.9	104.5 ± 2.1	NA	98.7 ± 6.2
	Superior	89.0 ± 6.4	97.8 ± 4.6	98.8 ± 7.1	97.7 ± 5.6	100.3 ± 5.4	99.3 ± 6.6	99.0 ± 8.1	105.0 ± 2.1	NA	98.8 ± 6.3
	Inferior	90.5 ± 6.4	98.0 ± 4.6	98.3 ± 7.1	97.1 ± 5.6	100.0 ± 5.4	98.4 ± 6.6	99.9 ± 8.1	104.5 ± 2.1	NA	98.5 ± 6.2

GCC = ganglion cell complex; OD = *Oculus Dexter* (right eye); OS = Oculus Sinister (left eye); NA = not applicable; RNFL = retinal nerve fiber layer.
